# Petunidin, a B-ring 5′-*O*-Methylated Derivative of Delphinidin, Stimulates Osteoblastogenesis and Reduces sRANKL-Induced Bone Loss

**DOI:** 10.3390/ijms20112795

**Published:** 2019-06-07

**Authors:** Masahiro Nagaoka, Toyonobu Maeda, Sawako Moriwaki, Atsushi Nomura, Yasumasa Kato, Shumpei Niida, Marlena C. Kruger, Keiko Suzuki

**Affiliations:** 1 Department of Pharmacology, School of Dentistry, Ohu University, Fukushima 963-8611, Japan; m-nagaoka@den.ohu-u.ac.jp; 2 Department of Oral Function and Molecular Biology, School of Dentistry, Ohu University, Fukushima 963-8611, Japan; t-maeda@den.ohu-u.ac.jp (T.M.); yasumasa-kato@umin.ac.jp (Y.K.); 3 Medical Genome Center, National Center for Geriatrics and Gerontology (NCGG), Aichi 474-8511, Japan; cyawa@ncvc.go.jp (S.M.); sniida@ncgg.go.jp (S.N.); 4 AKITAYA HONTEN Co., Ltd., Gifu 500-8471, Japan; a-nomura@akitayahonten.co.jp; 5 School of Health Sciences, College of Health, Massey University, Palmerston North 4442, New Zealand; M.C.Kruger@massey.ac.nz; 6 Department of Pharmacology, School of Dentistry, Showa University, Tokyo 142-8555, Japan

**Keywords:** petunidin, osteoblast, osteoclast, osteoporosis, anthocyanin, bone anabolism

## Abstract

Several lines of evidence suggest that oxidative stress is one of the key pathogenic mechanisms of osteoporosis. We aimed to elucidate the bone protective effects of petunidin, one of the most common anthocyanidins, considering its potent antioxidative activity. Petunidin (>5 μg/mL) significantly inhibited osteoclastogenesis and downregulated *c-fos*, *Nfatc1*, *Mmp9*, *Ctsk*, and *Dc-stamp* mRNA expression in RAW264.7 cells. Conversely, petunidin (>16 μg/mL) stimulated mineralized matrix formation and gene expression of *Bmp2* and *Ocn*, whereas it suppressed *Mmp13*, *Mmp2*, and *Mmp9* mRNA expression and proteolytic activities of *MMP13* and *MMP9* in MC3T3-E1 cells. Micro-CT and bone histomorphometry analyses of sRANKL-induced osteopenic C57BL/6J mice showed that daily oral administration of petunidin (7.5 mg/kg/day) increased bone volume to tissue volume (BV/TV), trabecular thickness (Tb.Th), trabecular number (Tb.N), the ratio of osteoid volume to tissue volume (OV/TV), osteoid thickness (O.Th), the ratio of osteoid surface to bone surface (OS/BS), the ratio of osteoblast surface to bone surface (Ob.S/BS), and the number of osteoblast per unit of bone surface (N.Ob/BS), and decreased trabecular separation (Tb.Sp), the ratio of eroded surface to bone surface (ES/BS), the ratio of osteoclast surface to bone surface (Oc.S/BS), and number of osteoclast per unit of bone surface (N.Oc/BS), compared to untreated mice. Furthermore, histological sections of the femurs showed that oral administration of petunidin to sRANKL-induced osteopenic mice increased the size of osteoblasts located along the bone surface and the volume of osteoid was consistent with the in vitro osteoblast differentiation and MMP inhibition. These results suggest that petunidin is a promising natural agent to improve sRANKL-induced osteopenia in mice through increased osteoid formation, reflecting accelerated osteoblastogenesis, concomitant with suppressed bone resorption.

## 1. Introduction

Osteoporosis, characterized by a significant decrease in bone mass and deterioration of the skeletal architecture [1], is thought to be one of the chronic and lifestyle-related diseases. Researchers have shown that increased oxidative stress was one of the key pathogenic mechanisms underlying both estrogen deficiency-induced [2,3,4] and age-related bone loss [5,6,7]. However, current anti-osteoporotic drugs, such as several bisphosphonates and anti-receptor activator of nuclear factor-κB ligand (RANKL) antibodies, are targeting osteoclast activity, rather than oxidative stress. Levis and Lagari reported that a good general nutritional status and adequate dietary consumption has a positive influence on bone health [8]. Therefore, a dietary approach, especially with regard to the inclusion of antioxidants, may be important for the primary prevention of bone loss, which may in turn lower the risk of fractures in later life.

Increasing evidence suggests that natural compounds in fruits and vegetables such as polyphenols have a wide range of biological and pharmacological effects and are now widely recognized as potential therapeutic candidates [9]. Among them, several flavonoids, a subgroup of polyphenols including hesperidin, quercetin, and luteolin, have been shown to prevent bone loss in ovariectomized mice [10,11,12]. Likewise, an anthocyanin-rich compound from blueberries, one of the flavonoids, was reported to be protective against bone loss in the ovariectomized rat model [13]. Furthermore, our previous study revealed that delphinidin, one of the major anthocyanidins, markedly inhibited the osteoclastic differentiation of RAW264.7 cells through suppression of *NF-κB, c-fos, and Nfatc1*, and prevented bone loss in osteoporotic mouse models [14].

Anthocyanins are a large flavonoid family of water-soluble red/blue/purple pigments in berry fruits and vegetables, and have been shown to provide a wide range of health-promoting benefits in human diseases, including cancer [15], diabetes [16], obesity [17], and cardiovascular disease [18], through most likely acting as antioxidants [17]. Among various types of flavonoids, anthocyanins have a higher antioxidant activity than other flavonoids, due to their positively charged oxygen atom [19] and their ability to scavenge free radicals by donating a hydrogen atom from a hydroxyl group as well as to support an unpaired electron. Despite their potent antioxidative activities, the health effects of anthocyanins in humans have been controversial due to their extremely low levels in the bloodstream. However, more detailed investigations performed recently revealed that not only the parent anthocyanin/anthocyanidin molecules but their metabolites including phenolic acids also act as potent antioxidants [9,20,21]. Additionally, anthocyanins can persist in circulation through enterohepatic recycling [20,21]. Thus, the bioavailability of anthocyanins may be far higher than suggested [9,20,21]. In fact, Welch et al. concluded that flavonoid intake, most prominently anthocyanin (median intake: 13.7 mg/day), had bone protective effects in their epidemiological study assessing the association between habitual flavonoid intake and bone mineral density in 3160 women [22].

In general, these phytochemicals have been reported to exert bone protective effects by inhibiting excessive bone resorption like most of the currently available synthetic agents including bisphosphonates. However, inhibiting bone resorption alone would not be fully effective for the treatment of estrogen deficiency- and age-related osteoporosis, in which suppressed bone formation is the crucial factor to be improved. Thus, an agent targeting both bone resorption and bone formation is needed. Lean et al. reported that antioxidants which increase tissue glutathione levels abolished ovariectomy-induced bone loss, where expression of TNFα, a target for NF-κB, was upregulated [2]. The NF-κB signaling pathway is critical for osteoclastogenesis. Conversely, NF-κB is also known to suppress osteoblastogenesis and bone formation [23,24,25]. Taken together with the fact that reactive oxygen species (ROS) can activate NF-κB, it is possible that the antioxidant petunidin could act as a bone anabolic agent.

In the present study, we investigated the effects of petunidin, a B-ring O-methylated derivative of delphinidin, on bone metabolism in vitro using mouse macrophage RAW 246.7 cells and preosteoblastic MC3T3-E1 cells, to examine osteoclastogenesis and osteoblastogenesis, respectively. Furthermore, we performed an in vivo study using a soluble RANKL (sRANKL)-induced osteopenic mouse model to evaluate its effectiveness in preventing bone loss over time when ingested daily.

## 2. Results

### 2.1. Effects of Anthocyanidins on Osteoclast Differentiation in RAW 264.7 Cells

**Figure 1:**
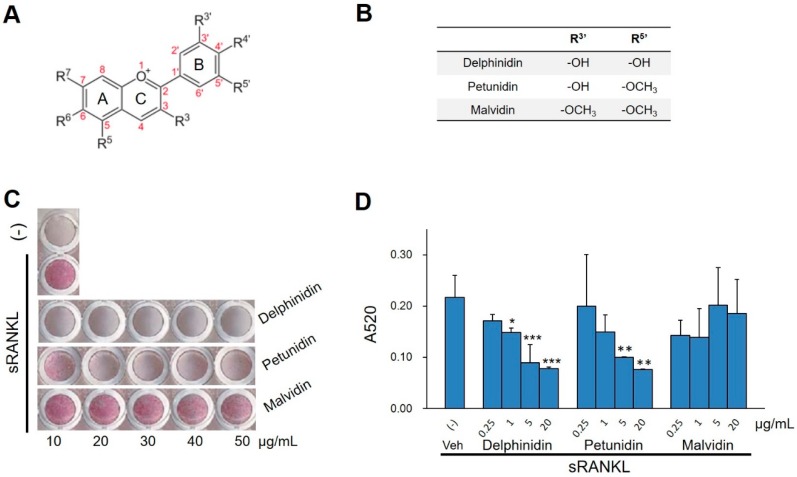
Effects of various anthocyanidins on osteoclastogenesis. (**A**) General structure of anthocyanidins. (**B**) Substituents in the R3′ and R5′ positions of the anthocyanidins used in the osteoclast formation assay. (**C**) Representative photographs of tartrate-resistant acid phosphatase (TRAP) staining in osteoclast cultures. RAW264.7 cells were pretreated for 1 h with increasing concentrations (10–50 µg/mL) of three anthocyanidins and cultured for 4 days in the presence of sRANKL (100 ng/mL). (**D**) Anti-osteoclastogenic activity of anthocyanidins was evaluated by the absorbance of the red-stained area per well at 520 nm on a spectrophotometer. Values are expressed in mean ± SD (*n* = 4). * *p* < 0.05, ** *p* < 0.01, *** *p* < 0.001 vs. vehicle (Veh).

We examined the effects of petunidin (10–50 μg/mL), a B-ring O-methylated delphinidin, on osteoclast formation using RAW264.7 cells in comparison to delphinidin (positive control for osteoclastogenesis inhibition) and malvidin, also one of the methylated anthocyanidins (Figure 1A,B). The number of tartrate-resistant acid phosphatase (TRAP)-positive multinucleated osteoclasts, differentiated from sRANKL-stimulated RAW264.7 cells, was markedly decreased by pretreatment with petunidin, although the inhibitory effect was weaker than that of delphinidin (Figure 1C). In contrast, no significant inhibitory effect of malvidin was found on osteoclast differentiation (Figure 1C). We next examined the dose-dependency of inhibitory effects on osteoclastogenesis with lower concentrations of these anthocyanidins. As shown in Figure 1D, 5–20 µg/mL of petunidin as well as 1–20 µg/mL of delphinidin inhibited osteoclastogenesis dose-dependently, whereas no dose-depend effect was observed for malvidin.

**Figure 2:**
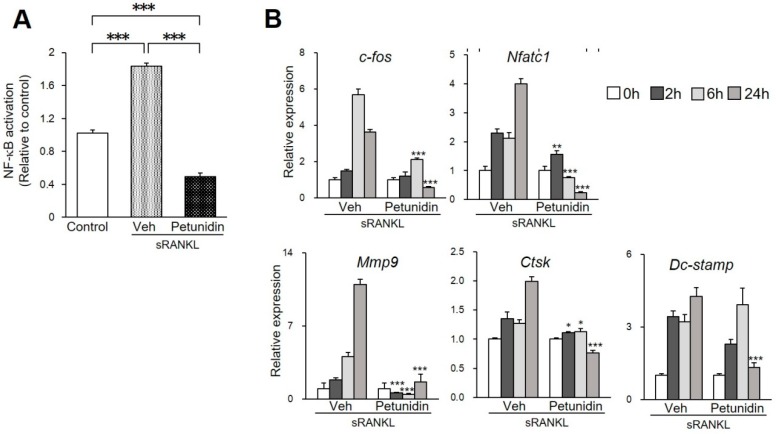
Effects of petunidin on the osteoclastogenesis. (**A**) NF-κB p65 subunit DNA-binding activity examined in RAW264.7 cells using the TransAM^®^ assay kit. (**B**) Quantitative real-time RT-PCR analyses of c*-fos, Nfatc1, Mmp9, CtsK* and *Dc-stamp* mRNA expression. The levels of mRNA expression were normalized with that of *Gapdh*. RAW 264.7 cells were cultured with or without 50 μg/mL of petunidin. Data are representative of three independent experiments and values are expressed in mean ± SD (*n* = 4). * *p* < 0.05, ** *p* < 0.01, *** *p* < 0.001 vs. vehicle (Veh) in the same culture period.

In order to know if petunidin inhibits osteoclastogenesis through suppressing NF-κB activation, we examined the NF-κB DNA-binding activity in RAW264.7 cells using the TransAM^®^ assay kit (Active Motif, Carlsbad, CA, USA). As shown in Figure 2A, 50 µg/mL petunidin significantly suppressed both sRANKL-induced and basal level transnucleation of the NF-κB p65 subunit and subsequent binding to DNA, suggesting that petunidin inhibits osteoclastogenesis at a transcriptional level to the same level as delphinidin [14]. Quantitative real-time RT-PCR (qPCR) analyses of RAW 264.7 cells showed that 50 µg/mL petunidin significantly inhibited sRANKL-induced upregulation of mRNA of *c-fos*, a key regulator of osteoclast-macrophage lineage determination [26] and *Nfatc1*, a master transcriptional factor located at the downstream cross-point of both NF-κB and c-Fos pathways in osteoclastogenesis [27,28] at 6 h and 24 h post-exposure. Furthermore, petunidin significantly downregulated the expression levels of *Mmp9* and *CtsK*, osteoclast marker genes, and that of *Dc-stamp*, a fusion protein gene for multinucleation of osteoclast precursor cells at 24 h of culture (Figure 2B).

### 2.2. Effects of Petunidin on Osteoblast Differentiation in MC3T3-E1 Cells

**Figure 3:**
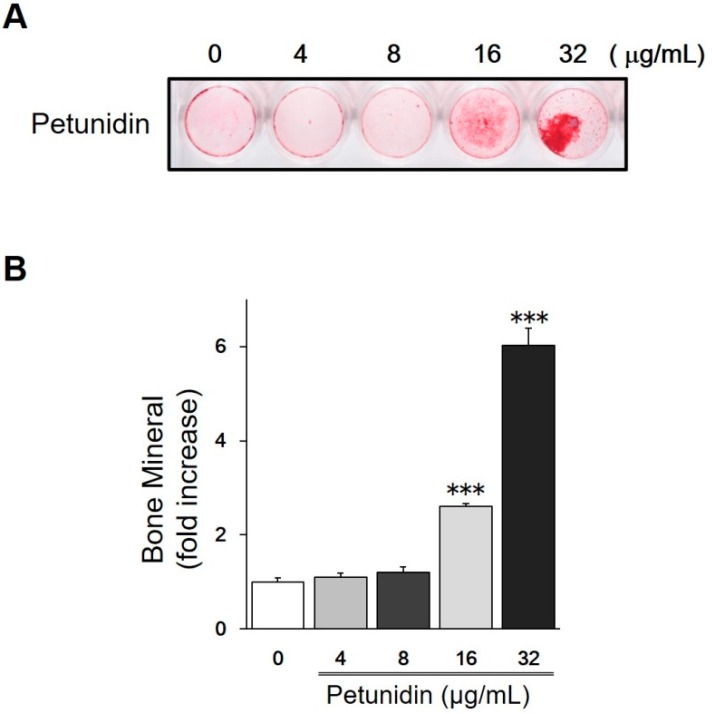
Effects of petunidin on in vitro osteoblastogenesis. (**A**) Representative photograph of mineralized nodule in MC3T3-E1 cells. Cells were treated with petunidin at the indicated concentrations for 16 days in differentiation media supplemented with 50 μg/mL ascorbate 2-phosphate, 10 mM β-glycerophosphate. At the end of incubation, cells were washed with PBS, fixed with 70% ethanol, and stained with Alizarin-red S. (**B**) Intensity of red stains of mineralized matrix measured using Molecular Imager^®^ (BioRad, Hercules, CA, USA). Values are expressed as mean ± SEM (*n* = 4). *** *p* < 0.001 vs. control (0 μg/mL of petunidin).

Petunidin (16 and 32 μg/mL, equivalent to 50 and 100 μM respectively) significantly stimulated multilayer proliferation of osteoblastic MC3T3-E1 cells, resulting in the increase in mineral deposition on day 16 of culture as shown in red with Alizarin red S staining (Figure 3).

**Figure 4:**
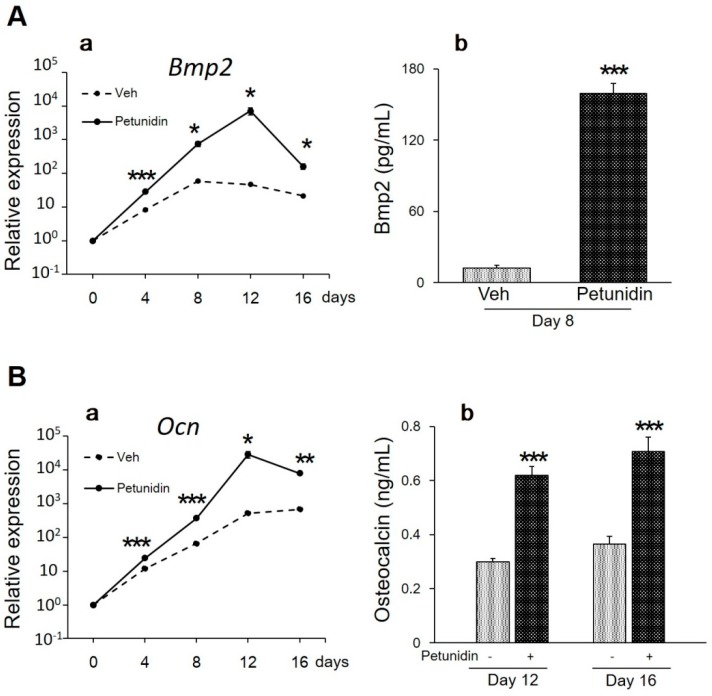
Effects of petunidin on in vitro osteoblast differentiation. (**A**) Expression levels of *Bmp2* mRNA (Figure 4Aa) and Bmp2 protein (Figure 4Ab) were analyzed by quantitative real-time RT-PCR (qPCR) and enzyme-linked immunosorbent assay (ELISA), respectively. (**B**) Expression levels of *Ocn* mRNA (Figure 4Ba) and Ocn protein (Figure 4Bb) were analyzed by qPCR and ELISA, respectively. MC3T3-E1 cells were cultured in the differentiation media with or without 32 μg/mL of petunidin. At the indicated time of incubation, total RNA was extracted, reverse-transcribed, and used for qPCR and conditioned media were harvested and used for ELISA. Values are expressed as mean ± SEM (*n* = 4). * *p* < 0.05, ** *p* < 0.01, *** *p* < 0.001.

Next, we investigated the effects of 32 μg/mL petunidin on the expression of osteoblastic differentiation markers by qPCR analysis and enzyme-linked immunosorbent assay (ELISA) in MC3T3-E1 cell cultures. As shown in Figure 4Aa, mRNA of bone morphogenetic protein (Bmp) 2, an anabolic signaling molecule in osteoblasts, was upregulated 12.7-fold, 151.8-fold and 7.5-fold compared with the vehicle on day 8, day 12, and day 16, respectively, by the petunidin treatment. Upregulated expression of Bmp2 was observed at protein level on day 8 as shown by ELISA (Figure 4Ab). Petunidin also upregulated osteocalcin (Ocn) mRNA, known as bone γ-carboxy glutamic acid protein and a marker of bone formation, 5.6-fold, 55.2-fold, and 11.6-fold compared with vehicle on day 8, day 12, and day 16, respectively (Figure 4Ba). Upregulation of Ocn by petunidin at protein level on day 12 and day 16 was also confirmed by ELISA (Figure 4Bb).

**Figure 5:**
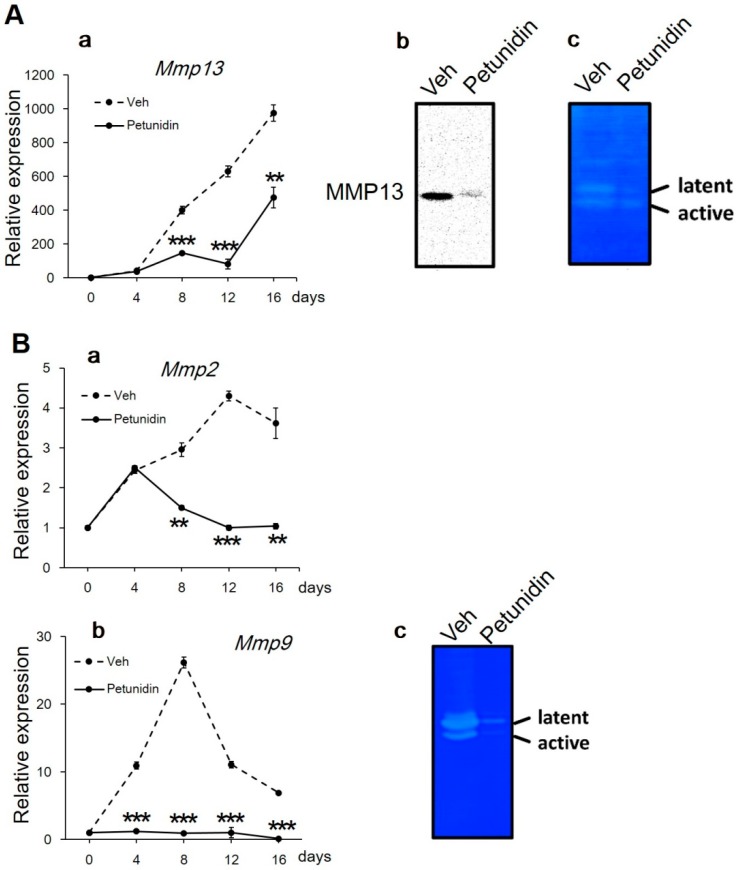
Effects of petunidin on ECM degradation. (**A**) Expression level of *Mmp13* mRNA (Figure 5Aa) was analyzed by qPCR. Expression of MMP13 protein was analyzed by western blotting (Figure 5Ab). In gel zymography was performed using the concentrated conditioned media harvested on day 16 in MC3T3-E1 cells cultured with or without 32 μg/mL petunidin. Samples were electrophoresed in 7.5% SDS-polyacrylamide gel containing 0.1% casein as a substrate, then stained with Coomassie Brilliant Blue R-250 after overnight incubation to detect a matrix-degrading activity (Figure 5Ac latent form and active form correspond to ~60 and ~48 kDa, respectively). (**B**) Expression levels of *Mmp2* and *Mmp9* mRNA (Figure 5Ba,b, respectively) were analyzed by qPCR. In gel zymography was performed in 7.5% SDS-polyacrylamide gel containing 0.1% gelatin as a substrate (Figure 5Bc, latent form and active form correspond to ~103 and ~86 kDa, respectively). Data in Figure 5Aa,Ba,Bb are expressed as mean ± SEM (*n* = 4). ** *p* < 0.01, *** *p* < 0.001.

Mature osteoblasts are characterized to produce and secrete a great amount of extracellular matrix (ECM) protein. In addition to this, since more ECM is needed to be produced in order to deposit increased amounts of mineral onto the matrix, we investigated the effects of 32 μg/mL petunidin on the gene expression of three major matrix metalloproteinase (MMP, comprising a large family of zinc-dependent endoproteinases capable of degrading ECM) [29], i.e., *Mmp13*, *Mmp2*, and *Mmp9*, encoding collagenase 3, gelatinase A, and gelatinase B, respectively, in MC3T3-E1 cells. As shown in Figure 5Aa,Ba, petunidin markedly downregulated mRNA expression levels of all three *Mmps* compared to untreated control, and the protein expression level of MMP13 (Figure 5Ab), the only molecule on which we performed western blot analysis in the present study. Furthermore, in gel zymography analyses showed that matrix-degrading activity of MMP13 and MMP9 was decreased by petunidin treatment for 16 days (Figure 5Ac,Bc, respectively). Collectively, these data suggested that petunidin suppressed the ECM degradation through downregulation of *Mmp* genes and/or matrix-degrading enzyme activities.

**Figure 6:**
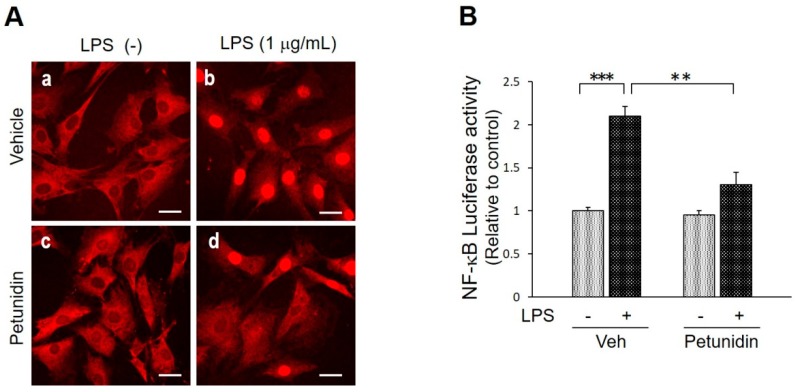
Effects of petunidin on NF-κB nuclear translocation. (**A**) Immunofluorescent staining of MC-3T3 cells for NF-κB p65. Cells were grown for 3 days with or without 32 μg/mL petunidin, then stimulated with 1 μg/mL lipopolysaccharide (LPS) for 1 h. Scale bars = 30 μm. (**B**) MC3T3-E1 cells were transfected with a mixture of pNF-κB-Luc and pGL4.75 [hRluc/CMV] for 4 h using Xfect^®^ Transfection Reagent (Clontech, Mountain View, CA, USA). Then the cells were pretreated with 32 μg/mL petunidin or vehicle for 24 h and then 1 μg/mL LPS was added only for the last 6 h to the end. Luminescent signals were measured using Dual-Luciferase^®^ Reporter Assay System (Promega, Madison, WI, USA). ** *p* < 0.01, *** *p* < 0.001.

In order to elucidate the mechanism through which petunidin stimulates the osteoblast differentiation and mineralized matrix formation, we examined the effects of petunidin on lipopolysaccharide (LPS)-induced NF-κB p65 nuclear translocation. Immunostaining for NF-κB p65, an active subunit of NF-κB, revealed that 32 μg/mL petunidin inhibited LPS-induced translocation of the p65 subunit into cell nuclei partially, which is clearly shown by the cytoplasmic, but not nuclei, staining (Figure 6Ad). Furthermore, NF-κB luciferase reporter assay revealed that transcriptional activity of NF-κB increased significantly by LPS stimulation. As shown in Figure 6B, petunidin significantly suppressed the LPS-induced upregulation of NF-κB transcription, supporting the immunofluorescence data (Figure 6A).

### 2.3. Protective Effects of Oral Administration of Petunidin against Bone Loss In Vivo

To determine the relevance of our in vitro findings of petunidin bioactivity to an in vivo model, we investigated the possible bone protective effects of petunidin in an osteopenic mouse model, a rapid (<50 h) bone loss model exhibiting a marked decrease in femoral trabecular bone mineral density (BMD) that is indistinguishable from that in ovariectomy [30]. Average bodyweights in the sRANKL-induced osteopenia model were 16.2 ± 0.6 g (14.9–17.1 g) and 18.1 ± 0.5 g (17.0–18.7 g), at the start and the end of the experiment, respectively. All the mice were healthy in terms of food and water intake, behavior, activity, general appearance, and within the anticipated normal body weight gain during the experiment. Organs (thymus, heart, lung, spleen, kidney, and liver) harvested at the end of the experiment showed no notable abnormalities and organ weight was within the range, which varied depending on their body size.

**Figure 7:**
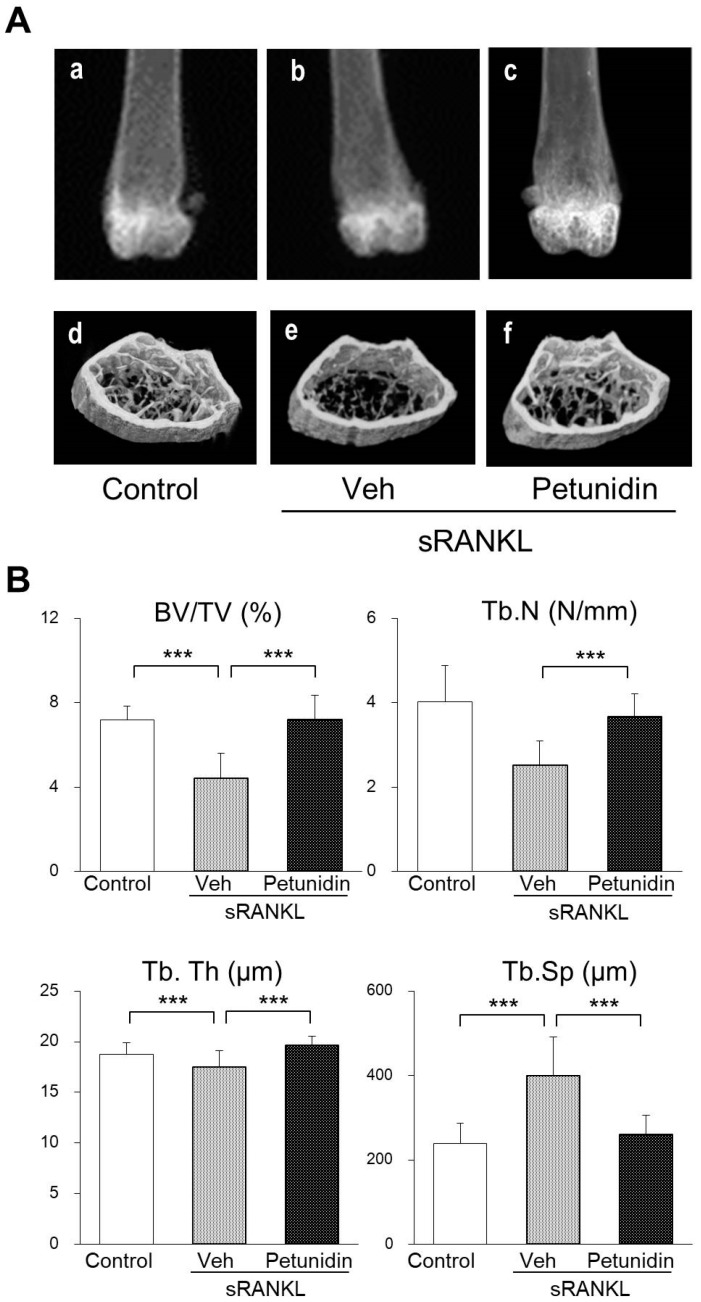
Effects of petunidin on bone loss in sRANKL-induced osteopenia mice. (**A**) Representative radiographs (Figure 7Aa–c) and micro-CT images (Figure 7Ad–f) of the distal femurs of intact mouse (control), sRANKL-induced osteopenia mouse (Veh), and petunidin (7.5 mg/kg/day)-treated sRANKL-induced osteopenia mouse. (**B**) Microarchitectural indices of second trabecular spongiosa of the distal femurs as measured by micro-CT on the areas shown in Figure 7Ad–f. Values are expressed as the mean ± SD. *** *p* < 0.001.

As shown in the three-dimensional (3D) images of the distal femurs constructed from microcomputed tomography (micro-CT) scans of sRANKL-induced osteopenia, the vacant area observed in the marrow cavity of vehicle group (Figure 7Ae) reduced in size and filled with trabeculae due to oral petunidin administration (Figure 7Af). Quantitative analyses by micro-CT showed that petunidin significantly increased bone volume to tissue volume (BV/TV), trabecular thickness (Tb.Th), trabecular number (Tb.N), and decreased trabecular separation (Tb.Sp), reflecting the increase in trabecular bone mass (Figure 7B). Additionally, 3D images demonstrated that some of the trabeculae observed in the petunidin group was thicker than that of the control mice, indicating that petunidin not only inhibited bone resorption but accelerated bone formation consistent with the in vitro findings (Figure 3 and Figure 4).

**Figure 8:**
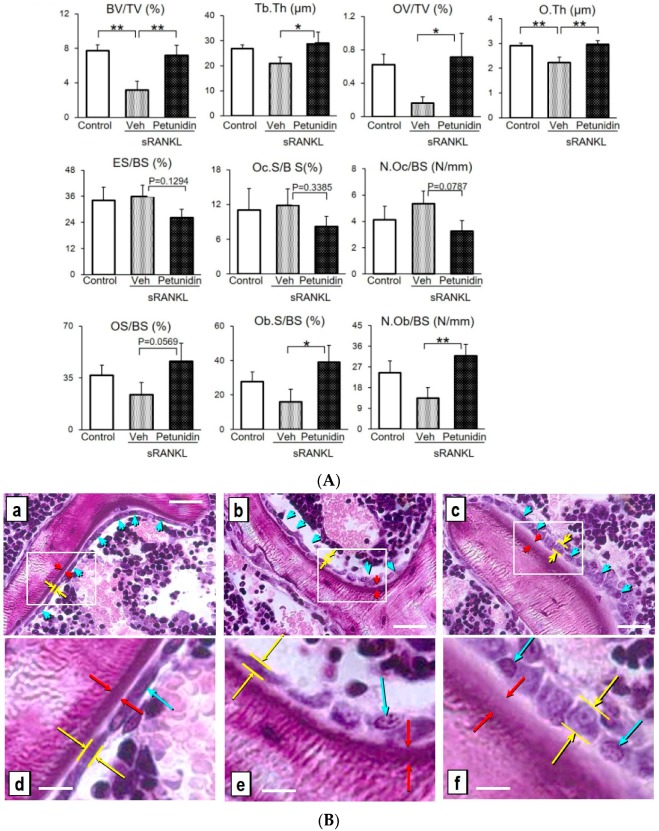
Histomorphometric analyses of protective effect of petunidin against bone loss in sRANKL-induced osteopenia mice. (**A**) Parameters were determined by morphometric analyses of second trabecular spongiosa of the distal femurs according to the methods of the ASBMR Histomorphometry Nomenclature Committee [31]. Values are expressed as the mean ± SD (*n* = 5). * *p* < 0.05, ** *p* < 0.01. (**B**) Representative histological frontal sections stained by Villanueva staining of distal femurs in intact mouse (Figure 8Ba,d; control), sRANKL-induced osteopenia mouse (Figure 8Bb,e; vehicle), and petunidin 7.5 (mg/kg/day)-treated sRANKL-induced osteopenia mouse (Figure 8Bc,f). Figures in the lower panel (d–f) correspond to the squares in the upper panel (a–c). Blue arrows represent the osteoblasts. Distance between two yellow arrows and that of red arrows represent height of osteoblast and osteoid thickness, respectively. Scale bars = 30 µm (Figure 8Ba–c), 10 µm ((Figure 8Bd–f).

In order to know whether the increase in trabecular bone mass by petunidin administration resulted only from suppressed bone resorption or in combination with accelerated bone formation, histomorphometric measurements were performed. Bone histomorphometry analyses on the distal femurs showed that petunidin significantly increased BV/TV and Tb.Th, compared to vehicle (Figure 8A), consistent with the micro-CT data (Figure 7B). Furthermore, petunidin administration significantly increased OV/TV and O.Th (Figure 8A), suggesting that bone formation was accelerated by petunidin. Bone formation parameters (OS/BS, Ob.S/BS and N.Ob/BS) were consistently and significantly increased by petunidin treatment. In contrast, major resorption-parameters (ES/BS, Oc.S/BS and N.Oc/BS) were decreased in the petunidin-treated group when compared to the vehicle group, although there were no statistically significant differences between groups (Figure 8A). Histological sections of the distal femurs of control- (Figure 8Ba,d), sRANKL alone- (Figure 8Bb,e) and (sRANKL + petunidin)-groups demonstrated that both osteoid thickness and height of the osteoblasts were increased by petunidin (Figure 8Bc,f).

## 3. Discussion

Among the phytochemicals that exhibit health promoting activity, anthocyanins, one of flavonoids, are known to be most commonly found in the diet such as berry fruits, vegetables, and wines. However, most studies have been performed using extracts of anthocyanin and anthocyanidin-containing fruits and berries or mixtures of different types of phytochemicals. McGhie pointed out that these naturally occurring anthocyanins could be modified during the processing and storage/shelf life of manufactured foods [9]. Additionally, these foods are also rich in vitamin C, which is known to exhibit health benefits by acting as a potent antioxidant. Therefore, the potential benefits of individual anthocyanins and anthocyanidins are uncertain. In order to clarify this, we have examined the effects of pure delphinidin aglycone in our previous study and demonstrated that delphinidin aglycone itself exerted bone protective effects both in vitro and in vivo [14]. The present study showed that purified petunidin aglycone, a B-ring 5′-*O*-methylated derivative of delphinidin which is found in many red berries including chokeberries, inhibited osteoclastogenesis dose-dependently in RAW 264.7 cells, whereas malvidin, the 3′, 5′-dimethoxy derivative of delphinidin, had no dose-dependent effects on osteoclastogenesis. Taken together with our previous finding that peonidin, 3′-*O*-methylated derivative of delphinidin, failed to inhibit osteoclastogenesis [14], the ortho-dihydroxyphenyl (both of 3′- and 4′-hydroxyl) structure seems to be critical for triggering anti-osteoclastogenic activity. On the other hand, Skates showed that the consumption of berries containing anthocyanins with enhanced methylation profiles (malvidin and petunidin) were more effective at reducing high fat diet-induced metabolic damage in mice [32]. However, the present study demonstrated that petunidin, a 5′-*O*-methylated derivative of delphinidin, was less potent than delphinidin, although petunidin downregulated expression levels of osteoclast-related mRNA, such as *c-fos*, *Nfatc1*, *Mmp9*, *CtsK*, and *Dc-stamp*, at least in part, through suppressing NF-κB activation, as well as delphinidin. Thus, the influences of the substitution in the 3′ and 5′ positions at the B-ring seem to be different among cell types and distinct bioactivities.

In contrast to the inhibitory effects on the osteoclast differentiation, petunidin significantly stimulated mineralized matrix formation and upregulated the expression of BMP2, an inducer of osteoblast differentiation [33], and Ocn, a marker of mineralization [34], at both gene and protein levels. Thus, our present study, showed that petunidin stimulated osteoblastogenesis concomitant with suppressed osteoclastogenesis through the mutual pathway in which downregulation of NF-κB signaling is involved. These effects of petunidin on two types of bone cells resulting in bone mass increase, are thought to be advantageous to a certain extent, over the anti-resorption agents including bisphosphonates. Moreover, although it might be possible that petunidin is a useful natural agent to the same extent as delphinidin despite less anti-osteoclastogenic activity, further investigation on the osteoblastogenic activity of delphinidin is required.

Furthermore, gene expression levels of collagenous ECM degrading enzymes such as MMP13, MMP2, and MMP9, which are produced by mature osteoblasts, were downregulated by petunidin treatment. Additionally, in gel analyses of MMP13 and MMP9 using individual substrates, i.e., casein and gelatin, demonstrated that bone matrix degradation activity was suppressed by petunidin. These effects of petunidin may in part be involved in the enhanced matrix accumulation observed in the MC3T3-E1 cell cultures. Since inhibitory effects of petunidin on *Mmp9* mRNA expression were also observed during the differentiation of RAW 264.7 cells, it is possible that these two phenomena work cooperatively to maintain bone mass through suppressing enzyme activities in osteoblasts as well as in osteoclasts in vivo. In support of our findings indicating petunidin can suppress matrix metalloproteinase (MMP) activity, Afaq et al. reported that anthocyanin-rich products protected the ECM of the skin by ameliorating the UVB-induced overexpression of various MMPs, such as collagenase (MMP1, corresponding to MMP13 in mice) and gelatinases (MMP2 and MMP9) [35]. Recently, Yu et al. reported resveratrol, a non-flavonoid polyphenol that acts as an antioxidant, protected osteoblasts from LPS-induced MMP2 production [36]. Another researcher reported that topical delivery of silk cocoon extract, one of the antioxidants, maintained the redox balance of the skin and prevented UV radiation-induced photoaging through downregulating MMP expression [37]. Additionally, Vincenti showed that NF-κB was involved in the process of activating matrix-degrading enzymes by proinflammatory cytokines [38]. Taken together, these results suggest that antioxidants protect various types of cells against matrix degradation by downregulating catabolic enzymes including MMPs through the inhibition of the NF-κB signaling pathway.

Consistent with the in vitro examinations, the increased number of osteoclasts under osteoporotic conditions was decreased by petunidin treatment (no statistical significance), resulting in prevention of bone loss in sRANKL-induced osteopenic mice. Mature osteoblasts are defined in vivo by their location along the bone surface and by their morphologic characteristics of plump cuboidal shape with large nuclei and actively producing and releasing specialized matrix proteins. Oral administration of petunidin to sRANKL-induced osteopenic mice increased the size of osteoblasts located along the bone surface, and the volume of osteoid, which was thought to be formed by utilizing larger amount of matrix proteins compared to control and sRANKL-treated (vehicle) mice. These observations in vivo were consistent with the in vitro osteoblast differentiation and MMP inhibition.

Bioavailability of anthocyanins had been known in the past to be extremely low due to very low circulating concentrations, especially detected as intact forms or aglycones [21]. Underestimation of circulating anthocyanin levels could be due to the use of various methodologies for the measurement of anthocyanins as flavylium cations, which are not detected after being metabolized to compounds, quinoniodal bases, hemiketals, and chalcones [9]. However, recent investigations revealed that the bioavailability of anthocyanins may be far higher than used to be suggested. Consistent with these reports, the present study, in which petunidin was administrated orally to mice, clearly showed that pure petunidin aglycone itself was absorbed from the stomach and/or intestine and distributed in the circulation as the effective molecules to improve bone metabolism. In support of our in vivo findings, Chen et al. showed that greater bone formation in blueberry-fed young rats was associated with an increase in osteoblast progenitors and osteoblast differentiation and reduced osteoclastogenesis through p38 MAPK β-catenin canonical Wnt signaling, and concluded that diet and nutritional status were critical factors that influenced bone development [39]. Hubert reviewed the studies regarding the impact of polyphenols on bone health and concluded that there was a positive association of high berry intake and higher bone mass [40]. Furthermore, Wallace reported that the lack of anthocyanin consumption had not been associated with any disorder because they are not essential nutrients; however, regular intake of anthocyanin-rich foods could confer protection against chronic diseases [41]. In the present in vivo study, the mice were administered with 7.5 mg/kg/day of petunidin. According to the concept of extrapolation of dose between species which is based on the body surface area, Nair and Jacob presented that the human equivalent dose can be estimated by dividing the mice dose by 12.3 [42]. According to this information, the human equivalent dose for petunidin used in the mice will be 0.61 mg/kg/day, which equals 36.58 mg/60 kg of person/day. Since this dose is in between 29 and 50 mg/person/day, the recommended daily anthocyanin intake in Japan [43] and China [44], respectively, daily consumption of petunidin may benefit the bone health in humans.

Meanwhile, based on the studies demonstrating the bioactivity of anthocyanins in mammalian cells [16,45,46], McGhie reported in a review that anthocyanins have the potential to interact with cells directly, although the studies were not proof of the absorption of anthocyanins into cells [9]. Likewise, although the present study showed that petunidin itself clearly exerted effects on bone metabolism both in vitro and in vivo, the possibility that petunidin acted indirectly through scavenging ROS produced outside the target cells, otherwise leading to NF-κB activation; is raised. We propose that this is one of the possible mechanisms by which petunidin functions in both types of bone cells simultaneously and improves bone metabolism. In support of this hypothesis, the literatures showed that ROS-induced activation of NF-κB stimulated osteoclastogenesis, and conversely, NF-κB activation induced inhibition of osteoblast differentiation [47] and bone formation in vivo [23,24,25].

In conclusion, the present study showed that petunidin aglycone, one of B-ring O-methylated derivatives of anthocyanidins, inhibited osteoclastogenesis in murine macrophage RAW 264.7 cells and stimulated mineralized matrix formation and downregulated matrix degrading enzymes in murine preosteoblastic MC3T3-E1 cells. Furthermore, oral administration of petunidin improved sRANKL-induced osteopenia in mice through increased osteoid formation, reflecting accelerated osteoblastogenesis, concomitant with suppressed bone resorption.

## 4. Materials and Methods

### 4.1. Anthocyanidins

Delphinidin chloride (C_15_H_11_O_7_Cl), petunidin chloride (C_16_H_13_O_7_Cl), and malvidin chloride (C_17_H_15_O_7_Cl) were purchased from Extrasynthése (Lyon, France).

### 4.2. Osteoclast Differentiation

RAW264.7 cells, a mouse macrophage cell line, were used as osteoclast precursor cells and maintained in α modified essential medium (α-MEM) supplemented with 10% fetal bovine serum (FBS) at 37 °C and 5% CO_2_. For osteoclast differentiation, cells were plated in a 96-well plate at a density of 4 × 10^3^ cells/well and stimulated with 100 ng/mL sRANKL for 4 days. For the inhibition study, cells were pre-incubated in α-MEM supplemented with vehicle or with various concentrations of anthocyanidins, 1 h before the addition of sRANKL (Oriental Yeast, Kyoto, Japan). To confirm multinucleated osteoclast formation, the cultured cells were fixed in 10% formalin for 3 min, and then stained with an osteoclast marker enzyme (TRAP) activity. The differentiation of osteoclasts was evaluated by measuring the intensity of TRAP staining at 520 nm using a spectrophotometer (SpectraMax M5; Molecular Devices, Sunnyvale, CA, USA).

Inhibitory effects of petunidin on sRANKL-induced NF-κB activation in RAW264.7 cells were examined according to the method previously described [14]. Briefly, cells were pretreated with petunidin for 1 h, and subsequently stimulated with sRANKL for 3 h. Then NF-κB p65 subunit DNA-binding activity of nuclear fractions, which were prepared using a Nuclear Extract Kit (Active Motif), were measured by using TransAM^®^ NF-κB p65 transcription factor assay kit (Active Motif). For qPCR, cells were incubated for the indicated time with or without petunidin in the presence of sRANKL. Total RNA was prepared using an Aurum^®^ Total RNA Mini Kit (Bio-Rad, Richmond, CA, USA). Then one microgram of total RNA from each sample was reverse-transcribed to cDNA with Ready-To-Go You-Prime First-Strand Beads (GE Healthcare, Piscataway, NJ, USA). cDNA samples were amplified by Thunderbird SYBR qPCR Mix (Toyobo, Osaka, Japan), with specific primers listed in Table 1, in a CFX96 Real-Time system (Bio-Rad). Expression levels were normalized to glyceraldehyde-3-phosphate dehydrogenase (*Gapdh*).

### 4.3. Osteoblast Differentiation

MC3T3-E1 cells, a clonal pre-osteoblastic cell line derived from newborn mouse calvaria, were maintained in a growth medium consisting of α-MEM supplemented with 10% FBS. For differentiation of the cells, we used a differentiation medium consisting of a growth medium supplemented with 50 μg/mL ascorbate 2-phosphate, 10 mM β-glycerophosphate, and 40 mM HEPES (pH 7.4). Mineralized matrix in the plates was stained with 40 mM Alizarin Red-S at pH 4.2 for 10 min at room temperature. The stained matrix was photographed, and the intensity of red stains was measured using Molecular Imager^®^ (BioRad). Total RNA was isolated with Isogen^®^ (Nippon Gene, Tokyo, Japan), and reverse-transcribed to synthesize cDNA with a High-Capacity cDNA Reverse Transcription Kit (Life Technologies, Rockville, MD). cDNA samples were amplified by SYBR Premix Ex *Taq* II (Takara Bio, Shiga, Japan) with specific primers listed in Table 1, in a Thermal Cycler Dice Real Time System (TP-870; Takara, Tokyo, Japan). The level of *Actb* (β-actin) mRNA was used as an internal control. BMP2 and OCN production were measured in the culture media harvested at the indicated time using BMP-2 Quantikine ELISA Kit (R&D Systems, Minneapolis, MN, USA) and Mouse Gla-Osteocalcin High Sensitive EIA Kit (Takara), respectively.

To investigate the effects of petunidin on ECM degradation, gene expression levels of *Mmp13* (collagenase 3), *Mmp2* (gelatinase A) and *Mmp9* (gelatinase B) were examined by qPCR using the specific primer sets listed in Table 1. Expression of MMP13 at protein level was detected by western blotting as previously described [48]. Briefly, MC3T3-E1 cells were incubated with or without petunidin for 8 days, then whole-cell lysates (10 μg/lane) were separated by 7.5% SDS-polyacrylamide gel electrophoresis (SDS-PAGE) and transferred onto Immobilon-P PVDF membranes (Merck Millipore, Billerica, MA, USA). Membranes were treated with rabbit polyclonal anti-MMP13 antibody (ab39012; Abcam, Cambridge, UK) followed by biotin-conjugated secondary antibody and avidin-conjugated horseradish peroxidase. Signals were detected with enhanced chemiluminescence reagent (Merck Millipore). To examine MMP activities in the culture media, we performed in gel zymography as previously described [49]. Briefly, concentrated culture media were separated by SDS-PAGE in 7.5% polyacrylamide gels containing 0.1% casein (MMP13) or 0.1% gelatin (MMP9). The gels were incubated in 50 mM Tris-HCl (pH7.5) buffer in the presence of 10 mM CaCl_2_ overnight at 37 °C. ECM degrading activities were visualized with Coomassie Brilliant Blue R250 staining and measured in a Molecular Imager^®^ (BioRad).

NF-κB nuclear translocation was investigated by immunohistochemistry and luciferase reporter assay in MC3T3-E1 cells. For immunohistochemical examination, cells were grown on a glass coverslip for 3 days in the absence or presence of 32 μg/mL petunidin and stimulated with 1 μg/mL LPS (*Escherichia coli* O111:B4, Merck KGaA, Darmstadt, Germany) for 1 h. Then, the cells were fixed with 2% paraformaldehyde and stained with goat polyclonal anti-NF-κB p65 antibody (C-20, sc-372-G, Santa Cruz Biotechnology, Dallas, TX, USA) followed by Alexa Fluor 594^®^-conjugated secondary antibody (Invitrogen, Carlsbad, CA, USA). Stained cells were examined under a laser scanning confocal microscope equipped with an optical laser unit and a scanning unit (FV1000; Olympus Optical, Tokyo, Japan). For luciferase reporter assay, cells were transfected with a mixture of pNF-κB-Luc (Agilent Technologies, Santa Clara, CA, USA) and pGL4.75 [hRluc/CMV] (Promega) using Xfect^®^ Transfection Reagent (Clontech). Then the cells were incubated with or without 32 μg/mL petunidin for 24 h and 1 μg/mL LPS was added only for the last 6 h to the end. Luminescent signals were measured using Dual-Luciferase^®^ Reporter Assay System (Promega).

### 4.4. In Vivo Experiments Using sRANKL-Induced Osteopenic Mouse Model

To assess the protective effect of petunidin on bone loss in vivo, we used a sRANKL-induced osteopenic mouse model, which was established by Yasuda and his colleagues [30]. Seven-week-old female C57BL/6J mice were purchased from CLEA Japan (Tokyo, Japan). The mice were divided into three groups: Control (*n* = 6), sRANKL-induced osteopenic mice (vehicle, *n* = 6) and 7.5 mg/kg/day petunidin-treated osteopenic mice (petunidin, *n* = 6). The mice were intraperitoneally injected with sRANKL (1.0 mg/kg; Oriental Yeast, Kyoto, Japan) twice at an interval of 2 days. For petunidin-treated mice, petunidin oral administration via a flexible plastic tube fitted with a stainless blunted end needle gavage (0.2 mL/mouse) started 3 days before the first injection of sRANKL and continued for 14 days while being dosed with petunidin daily. Control and vehicle mice received the same volume of water. All mice were housed in an animal room (temp, 22 ± 2 °C; humidity, 50%; light/dark cycle, 12 h) with free access to food and water. All animal experiments were conducted in compliance with the commonly-accepted ‘3Rs’—Replacement, Reduction, Refinement—according to the protocol which was approved by the “Animal Experimental Committees of Showa University” (project identification code: 12071, issued on 10/09/2012) and “Experimental Animal Center in Ohu University” (project identification code: 2017-7, issued on 01/04/2017 and 2018-9, issued on 01/04/2018).

### 4.5. Bone Analyses

Bone morphometric parameters and microarchitectural properties of the femur were determined using a micro-CT system (inspeXio SMX-90CT; Shimadzu, Kyoto, Japan) as previously described [14]. For quantitative analysis of bone structural indices, the ratio of bone volume to tissue volume (BV/TV), trabecular thickness (Tb.Th), trabecular number (Tb.N), and trabecular separation (Tb.Sp) were determined according to the guidelines for assessment of bone microstructure in rodents using micro-computed tomography [50] using TRI/3D-BON software. For histomorphometric analysis, femurs were fixed in 70% ethanol and stained with Villanueva-Goldner bone stain (Wako Pure Chemical Industries, Osaka, Japan) and then embedded in glycolmethacrylate without decalcification. Frontal sections of the distal end of femurs were observed using a fluorescent microscope (BX-53, Olympus Optical). The results of the bone histomorphometric analyses, BV/TV, Tb.Th, the ratio of osteoid volume to tissue volume (OV/TV), osteoid thickness (O.Th), the ratio of eroded surface to bone surface (ES/BS), the ratio of osteoclast surface to bone surface (Oc.S/BS), the number of osteoclast per unit of bone surface (N.Oc/BS), the ratio of osteoid surface to bone surface (OS/BS), the ratio of osteoblast surface to bone surface (Ob.S/BS), and the number of osteoblast per unit of bone surface (N.Ob/BS) are expressed according to the methods of the ASBMR Histomorphometry Nomenclature Committee [31].

### 4.6. Statistical Analysis

Statistical analyses were performed using unpaired two-sample *t*-test for Figure 2B, Figure 4, and Figure 5Aa,Ba,b, and using one-way analysis of variance (ANOVA) for comparison among all groups for Figure 1D, Figure 2A, Figure 3B, Figure 6B, Figure 7B, and Figure 8B. The Tukey-HSD (honestly significant difference) test was used for post hoc pair-wise comparisons after the ANOVA. All statistical analyses were performed using KaleidaGraph. A *p*-value less than 0.05 was considered statistically significant.

## Figures and Tables

**Table 1:** Primer sequences for RT-qPCR.

Genes	Proteins (Abbreviations)	Primer Sequences		Product (bp)
c-fos	c-Fos	Forward:	5′-GTC CGG TTC CTT CTA TGC AG-3′	128
		Reverse:	5′-TAA GTA GTG CAG CCC GGA GT-3′	
Nfatc1	Nuclear factor of activated T cells, cytoplasmic 1 (NFATC1/NFAT2)	Forward:	5′-CCC GTT GCT TCC AGA AAA TA -3′	94
		Reverse:	5′-TCA CCC TGG TGT TCT TCC TC -3′	
Mmp9 (OC*)	Matrix metalloproteinase 9 (MMP9)	Forward:	5′-CCA GGA TAA ACT GTA TGG CT-3′	121
		Reverse:	5′-ACA GGA AGA GTA CTG CTT GC-3′	
Ctsk	Cathepsin K (CTSK)	Forward:	5′-GGG AGA CAT GAC CAG TGA AG-3′	73
		Reverse:	5′-ACT GTA GGA TCG AGA GGG AG-3′	
Dc-stamp	Dendritic cell specific transmembrane protein (DC-STAMP)	Forward:	5′-AAA ACC CTT GGG CTG TTC TT-3′	115
		Reverse:	5′-GTT CAT GGA GGA GAT GAG CC-3′	
Gapdh	Glyceraldehyde-3-phosphate dehydrogenase (GAPDH)	Forward:	5′-AAT GGT GAA GGT CGG TGT G-3′	226
		Reverse:	5′-GAA GAT GGT GAT GGG CTT CC-3′	
Bmp2	Bone morphogenetic protein 2 (BMP2)	Forward:	5′-TGA CTG GAT CGT GGC ACC TC-3′	112
		Reverse	5′-CAG AGT CTG CAC TAT GGC ATG GTT A-3′	
Ocn	Osteocalcin (OCN)	Forward:	5′-GTG AGC TTA ACC CTG CTT GT-3′	96
		Reverse:	5′-AGC ACA GGT CCT AAA TAG TGA TAC C-3′	
Mmp13	Matrix metalloproteinase 13 (MMP13)	Forward:	5′-TCC CTG GAA TTG GCA ACA AAG-3′	120
		Reverse:	5′-GCA TGA CTC TCA CAA TGC GAT TAC-3′	
Mmp2	Matrix metalloproteinase 2 (MMP2)	Forward:	5′-AAC GGT CGG GAA TAC AGC AG-3′	125
		Reverse:	5′-GTA AAC AAG GCT TCA TGG GGG -3′	
Mmp9 (OB**)	Matrix metalloproteinase 9 (MMP9)	Forward:	5′-GCC CTG GAA CTC ACA CGA CA-3′	85
		Reverse:	5′-TTG GAA ACT CAC ACG CCA GAA G-3′	
Actb	β-actin	Forward:	5′-CAT CCG TAA AGA CCT CTA TGC CAA C-3′	171
		Reverse:	5′-ATG GAG CCA CCG ATC CAC A-3′	

OC*; murine macrophage cell line RAW264·7 used for osteoclastogenesis. OB**; murine pre-osteoblastic cell line MC3T3-E1 used for osteoblastogenesis.
